# Approaches to Understanding Mechanisms of Dentilisin Protease Complex Expression in *Treponema denticola*

**DOI:** 10.3389/fcimb.2021.668287

**Published:** 2021-05-18

**Authors:** M. Paula Goetting-Minesky, Valentina Godovikova, J. Christopher Fenno

**Affiliations:** Department of Biologic and Materials Sciences & Prosthodontics, School of Dentistry, University of Michigan, Ann Arbor, MI, United States

**Keywords:** spirochete, protease, mutagenesis, periodontitis, dentilisin

## Abstract

The oral spirochete *Treponema denticola* is a keystone periodontal pathogen that, in association with members of a complex polymicrobial oral biofilm, contributes to tissue damage and alveolar bone loss in periodontal disease. Virulence-associated behaviors attributed to *T. denticola* include disruption of the host cell extracellular matrix, tissue penetration and disruption of host cell membranes accompanied by dysregulation of host immunoregulatory factors. *T. denticola* dentilisin is associated with several of these behaviors. Dentilisin is an outer membrane-associated complex of acylated subtilisin-family PrtP protease and two other lipoproteins, PrcB and PrcA, that are unique to oral spirochetes. Dentilisin is encoded in a single operon consisting of *prcB*-*prcA-prtP*. We employ multiple approaches to study mechanisms of dentilisin assembly and PrtP protease activity. To determine the role of each protein in the protease complex, we have made targeted mutations throughout the protease locus, including polar and nonpolar mutations in each gene (*prcB*, *prcA*, *prtP*) and deletions of specific PrtP domains, including single base mutagenesis of key PrtP residues. These will facilitate distinguishing between host cell responses to dentilisin protease activity and its acyl groups. The boundaries of the divergent promoter region and the relationship between dentilisin and the adjacent iron transport operon are being resolved by incremental deletions in the sequence immediately 5’ to the protease locus. Comparison of the predicted three-dimensional structure of PrtP to that of other subtilisin-like proteases shows a unique PrtP C-terminal domain of approximately 250 residues. A survey of global gene expression in the presence or absence of protease gene expression reveals potential links between dentilisin and iron uptake and homeostasis in *T. denticola*. Understanding the mechanisms of dentilisin transport, assembly and activity of this unique protease complex may lead to more effective prophylactic or therapeutic treatments for periodontal disease.

## Introduction

**Figure 1:**
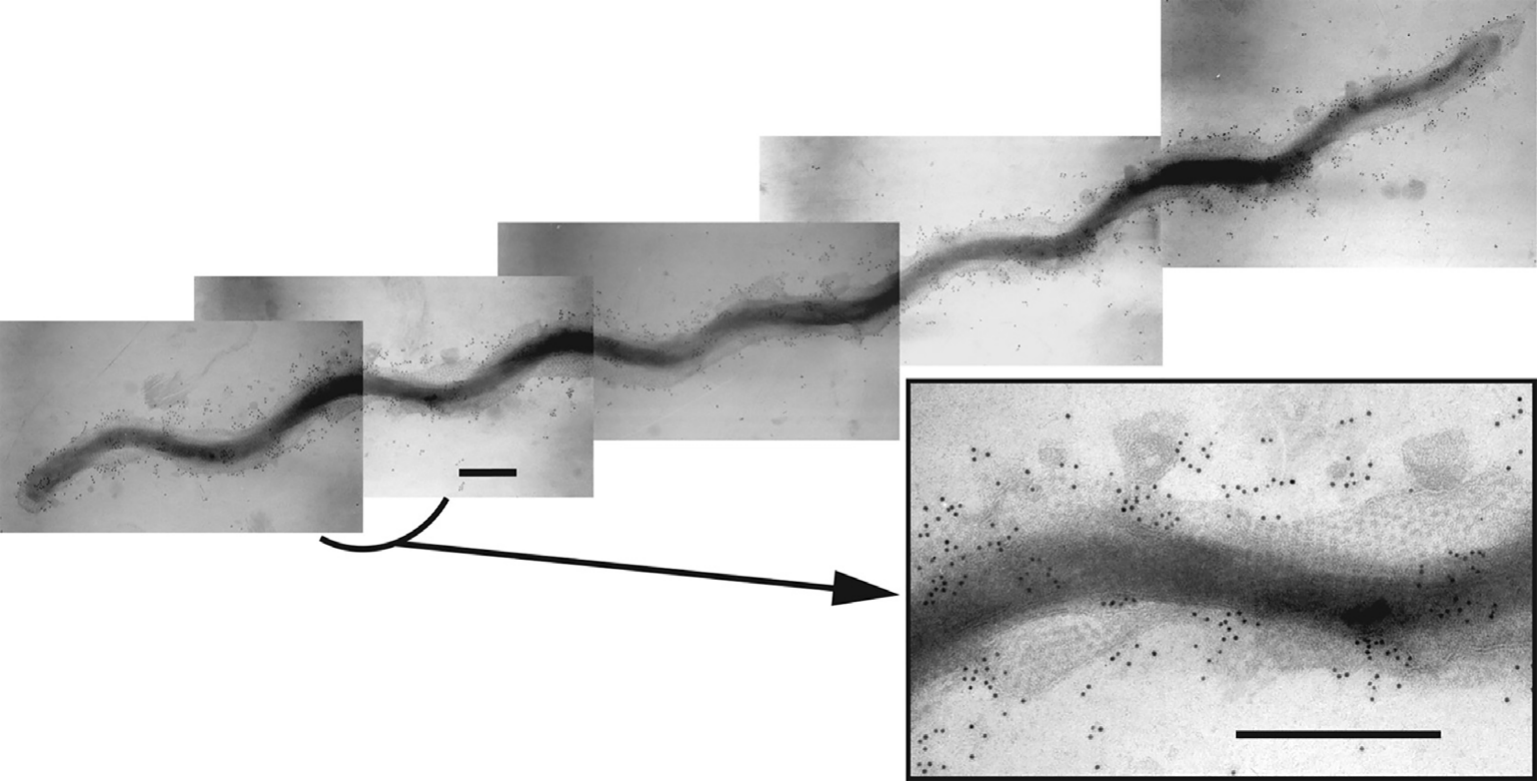
Composite immunogold transmission electron micrograph showing a *T. denticola* ATCC 35405 dividing cell labelled with rabbit immunoglobulins raised against the dentilisin protease complex followed by gold-conjugated anti-rabbit IgG, as reported previously (Grenier et al., 1990). The hexagonal array of the Msp complex is visible in the outer membrane. The inset shows an enlargement of the indicated region. Bar: 300 nm. [reprinted with permission from (Fenno, 2011)].

*Treponema denticola* (**Figure 1**) is an oral spirochete that is a periodontal pathogen capable of inducing extensive host tissue damage (e.g. chronic periodontitis) (Loesche, 1988) in association with other members of a complex polymicrobial oral biofilm (Socransky et al., 1998; Hajishengallis, 2014). Degradation of host extracellular matrix components and host immunoregulatory factors (Uitto et al., 1988; Uitto et al., 1995; McDowell et al., 2011) are some of the virulence-associated behaviors exhibited that have established *T. denticola* as the model organism for studying both *Treponema* biology and host interactions in periodontal disease. *T. denticola* dentilisin is expressed from an operon comprised of genes *prcB*, *prcA* and *prtP* (Ishihara et al., 1996; Lee et al., 2002; Godovikova et al., 2010). Post-translational processing and interactions among the expressed proteins result in the formation of an active protease complex on the surface of *T. denticola* (Lee et al., 2002; Godovikova et al., 2011). We are using multiple approaches to understand the mechanism of PrtP activity in *T. denticola* ATCC 35405 and its associations with other proteins.

Severe periodontitis lesions typically contain elevated levels of spirochetes, preferentially localized in intimate contact with tissue at the deepest regions of lesions (Sela, 2001). Spirochetes have both direct and indirect cytopathic effects contributing to both bacterial tissue penetration and destructive host responses (Lux et al., 2001; Chi et al., 2003). In active periodontitis, host proteases cleave extracellular matrices, releasing fragments of fibronectin (FN) into the local microenvironment. A specific FN fragment pattern in gingival crevicular fluid (GCF) is a marker of periodontal disease status (Kapila et al., 1996; Huynh et al., 2002). Moreover, certain FN fragments (but not intact FN) induce apoptosis and suppress osteoblast differentiation of periodontal ligament cells (PDL), a key consequence of periodontal dysbiosis (Dai et al., 2005; Ghosh et al., 2010). Mechanism(s) of bioactive FN fragments generation, including relative contributions of bacterial and host proteases, have not been established. Bacterial factors that disrupt periodontal homeostasis are of prime interest as mediators of chronic local and systemic inflammatory challenge by oral organisms and as possible therapeutic targets.

*T. denticola*, the most readily cultivable oral spirochete, is the model organism for spirochete-host interactions in periodontitis. Two *T. denticola* outer membrane (OM) protein complexes directly affect host cells: the surface-expressed PrtP lipoprotein protease complex (dentilisin) and the oligomeric major surface protein (Msp). Besides its role in nutrient acquisition, dentilisin contributes to tissue penetration and cleaves proteins involved in both innate and acquired immunity (Chi et al., 2003; Miyamoto et al., 2006; McDowell et al., 2011; Ruby et al., 2018). Dentilisin is required for native expression of Msp (Ishihara et al., 1996; Fenno et al., 1998), a prominent surface-expressed protein that binds FN, has cytotoxic pore-forming activity and disrupts intracellular cytoskeletal and calcium responses (Wang et al., 2001; Visser et al., 2011). To determine their roles in microbe-host interactions, it is necessary to understand the interdependent expression of these protein complexes on the surface of the spirochete. Recent advances in *T. denticola* molecular biology make feasible the analysis of these immunomodulatory and cytopathic behaviors (Li et al., 2015). A major focus of research in our laboratory is characterization of expression, processing and assembly of the dentilisin protease complex on the *T. denticola* outer membrane surface. These studies are designed to support ongoing research into the cytopathic effects and responses (at both the cellular and tissue levels) to *T. denticola* and specifically to its dentilisin complex. This includes characterization of two very different features of dentilisin: the role of its proteolytic activity and the role of its acyl components in inducing these responses. Here we report on recent advances in dentilisin characterization within the context of prior work by us and others, in order to provide a broad picture of the current state of knowledge of the composition and expression of this unique acylated protease complex.

## Materials and Methods

### Bacterial Strains and Growth Conditions

*T. denticola* strains were grown as previously described (Fenno, 2005) under anaerobic conditions in NOS or TYGVS broth or semisolid medium containing 0.8% Noble agar, supplemented with erythromycin (Em; 40 µg ml^-1^) or kanamycin (Km; 25 µg ml^-1^) as appropriate. Purity of spirochete cultures was monitored by darkfield microscopy. *E. coli* strains were grown in LB medium supplemented with Km (50 µg/ml) or carbenicillin (Cb; 50 µg/ml) as appropriate. Routine cloning was done in *E. coli* JM109 or JM110 (Yanisch-Perron et al., 1985).

### Construction of Plasmids for Mutagenesis Studies

All plasmids were constructed using either restriction fragments of previously verified plasmids or PCR products generated with high-fidelity DNA polymerases: Phusion (New England Biolabs, Beverly, MA) or Taq-HF (Invitrogen, Carlsbad, CA). Mutation constructs were made in *E. coli* plasmid vector pGEM-T-Easy or pBLUEScript SK-. Plasmid constructs were verified by DNA sequencing at the University of Michigan DNA Sequencing Core Facility and analyzed using DNASTAR sequence analysis software (DNASTAR Inc., Madison, WI).

To delete the *prtP* C-terminal domain, the *ermB* gene cassette (Goetting-Minesky and Fenno, 2010) was cloned between DNA fragments consisting of (1) a *prtP* fragment lacking the 259-residue C-terminal domain and (2) a fragment 3’ to *prtP* including part of TDE0765, resulting in plasmid pCF998. This plasmid, digested with restriction enzymes to release the vector sequence, was used to transform *T. denticola* by allelic replacement mutagenesis as described below.

To introduce a single base change resulting in the desired Ser^447^➔Ala mutation in one of the three *prtP* active site residues, *prtP* DNA encoding residues 230-766 was subjected to site-directed mutagenesis using the QuickChange XL kit (Stratagene) according to the manufacturer’s protocol. The mutated *prtP* DNA was then cloned into pSY119 (Lee et al., 2002) such that it replaced the wild type *prtP* sequence on that plasmid. Subsequently, DNA including the 5’ region of TDE0765 (3’ to prtP) was amplified from *T. denticola* genomic DNA and ligated to the prior construct. The resulting construct (pCF649) carries the 3’ region of *prcA*, the *prtP* coding sequence carrying the Ser^447^➔Ala mutation and DNA 3’ to *prtP* through the 5’ region of TDE0765. Lastly, by a combination of overlap extension PCR (Horton et al., 1989; Hilgarth and Lanigan, 2020) and FastCloning (Li et al., 2011), a fragment consisting of the *aphA2* gene encoding Km^R^ preceded by a RBS sequence was cloned directly 3’ to the *prtP* stop codon. The resulting plasmid (pCF1024), digested with restriction enzymes to release the vector sequence, was used to transform *T. denticola* by allelic replacement mutagenesis as described below.

### Allelic Replacement Mutagenesis of *T. denticola*

Defined isogenic mutants were constructed as described previously (Li et al., 1996; Fenno et al., 1998), by electroporation of *T. denticola* with linear DNA fragments consisting of the selectable *ermB* or *aphA2* cassette cloned between DNA fragments flanking the mutagenesis target. The C-terminal mutant was constructed in the wild type strain *T. denticola* ATCC 35405 by electroporation with linearized pCF998 and selection for Em^R^. The active site mutant was constructed in the PrtP C-terminal deletion strain *T. denticola* CF1010 (**Table 1**) by electroporation with linearized pCF1024 and selection for Km^R^. Mutations in *T. denticola* were confirmed by DNA sequencing at the University of Michigan DNA Sequencing Core Facility and analyzed using DNASTAR sequence analysis software (DNASTAR Inc., Madison, WI).

**Table 1:** Dentilisin phenotypes of *T. denticola* mutant strains.

Strain/Genotype	Dentilisin phenotype						
Strain (mutation)(source)	PrcB	PrcA1	PrcA2	PrtP-N	PrtP	SAAPFNA*	Msp
35405 wild type (Chan et al., 1993)	+	+	+	+	+	+	+
MHE Δ*msp* (Fenno et al., 1998)	+	+	+	(+) nd	+	+	Ø
CKE Δ*prcA-prtP* (Fenno et al., 1998)	(+) nd	+ (truncated, uncleaved)		Ø	Ø	Ø	Ø
PNE Δ*prcA* (polar insertion) (Lee et al., 2002)	(+) nd	Ø	Ø	(Ø) nd	Ø	Ø	(Ø) nd
CCE Δ*prtP* (Lee et al., 2002)	(+) nd	+ (uncleaved)		(Ø) nd	Ø	Ø	Ø
P0760 Δ*prcB* (polar) (Bian et al., 2005)	Ø	Ø	Ø	(Ø) nd	Ø	Ø	Ø
CF417 *prcB-*6xHis (polar) (Godovikova et al., 2010)	+	Ø	Ø	Ø	Ø	Ø	Ø
CF499 *prcB-*6xHis (non-polar) (Godovikova et al., 2010)	+	nd	+	(+) nd	+	nd	nd
CF522 Δ*prcB* deletion (non-polar) (Godovikova et al., 2010)	Ø	+ (uncleaved)		(Ø) nd	Ø	Ø	+
CF646 *prtP-*6xHis (Goetting-Minesky et al., 2012)	+	+	+	(+) nd	+	+	+
CF547 Δ*prcA* polar insertion (Goetting-Minesky and Fenno, 2010)	(+) nd	Ø	Ø	(Ø) nd	Ø	(Ø) nd	Ø
CF548 Δ*prcB* polar insertion (Goetting-Minesky and Fenno, 2010)	(Ø)	Ø	Ø	(Ø) nd	Ø	(Ø) nd	Ø
CF942 Δ*prcA* non-polar deletion (unpublished)	+	Ø	Ø	(+) nd	+**	nd	Ø
CF1010 *prtP* ΔC-term (this study)	+	+ (uncleaved)		Ø	Ø	Ø	Ø
CF1031 *prtP*: Ser^447^Ala (this study)	+	+	+	++++	+***	Ø	+

*: Cleavage of N-succinyl-Ala-Ala-Pro-Phe p-nitroanilide substrate.

**: not processed.

***: PrtP with Ser^447^➔Ala point mutation. Mutants have been made in each of the protease genes. Genes downstream of polar insertion of an antibiotic resistance cassette in the protease operon are not expressed. Deletions within *prtP* inhibit activity of PrtP and affect processing of PrcA. Results are from western blot analyses using specific primary antibodies and SAAPFNA assays. Symbols: + (expressed); Ø (not expressed); nd (no data, with or without predicted result).

### Protein Gel Electrophoresis and Immunoblotting

SDS-PAGE and western immunoblotting were done as described previously (Fenno et al., 1996). Total cell lysates of *T. denticola* strains were heated at 95°C for 5 min, then separated by SDS-PAGE and transferred to nitrocellulose membranes, which were then probed with rabbit polyclonal antibodies raised against various components of the dentilisin locus (PrcB, PrcA1, PrcA2, PrtP, PrtP-N) followed by horseradish peroxidase (HRP)-conjugated goat anti-rabbit IgG or (HRP)-conjugated goat anti-rat IgG (Thermo Scientific, Rockford, IL) as appropriate. Protein bands of interest were visualized using SuperSignal West Pico chemiluminescent substrate (Thermo Scientific) and a G:Box imaging system (Syngene, Frederick, MD).

### Predictive Bioinformatic Analysis of PrtP Structure

Three-dimensional protein structure models were generated from primary amino acid sequences using the public I-TASSER server at https://zhanglab.ccmb.med.umich.edu/. The I-TASSER algorithm consists of four general steps: threading template identification, iterative structure assembly simulation, model selection and refinement, and structure-based function annotation to generate rigorous structure models in the absence of experimentally determined protein structure (Yang et al., 2015). The amino acid sequence of the mature, active PrtP protein (residues 159-766) was compared with that of mature subtilisin from *Bacillus subtilis* subsp. *subtilis* [GenBank: AEP90133.1; (Earl et al., 2012)]. Protein structure models generated in I-TASSER were labeled, scaled and annotated using the Protean 3D component of the Lasergene Molecular Biology Suite (DNASTAR Inc., Madison, WI).

### Protease Activity Assay

Hydrolysis of chromogenic substrate succinyl-Lalanyl-L-alanyl-L-prolyl-L-phenylalanine-p-nitroanilide (SAAPFNA) by *T. denticola* parent and mutant strains was conducted as described previously (Fenno et al., 2001; Goetting-Minesky et al., 2012). For assays, four-day cultures were adjusted to an A_600_ of 0.25 in TYGVS broth. TYGVS broth alone served as a blank control. The experiment was conducted three times with triplicate samples. Protease activity of individual strains, assayed by change in absorbance at 405 nm, is expressed as a percentage relative to the SAAPFNA activity of strain 35405. Error bars represent the standard error of three independent experiments with triplicate samples.

### Transcriptome Analysis by RNAseq

*T. denticola* strains were grown in NOS medium and harvested at early log phase (OD_600_ of approximately 0.2). Total RNA was isolated, treated with DNAse and assayed for quality and concentration as described previously (Saraithong et al., 2020). Subsequent steps in RNAseq analysis were conducted at the University of Michigan DNA Sequencing Core Facility. Ribosomal RNA was depleted from purified RNA samples using a RiboZero rRNA Removal Kit for Gram-negative bacteria (Illumina, San Diego, CA, USA). RNA integrity was determined using an Agilent 2100 Bioanalyzer (San Diego, CA, USA). Only samples with RNA Integrity Number greater than 8 were used for construction of cDNA libraries. Sequencing was performed on a HiSeq 4000 system instrument (Illumina) using the clustering and sequencing reagents provided by Illumina. Paired-end raw reads in FASTQ format were trimmed using the Trimmomatic algorithm (Bolger et al., 2014). Trimmed reads for each sample and end were assessed for quality using FastQC (https://www.bioinformatics.babraham.ac.uk/projects/fastqc/). Reads were aligned to the reference *T. denticola* ATCC 35405 genome. Gene expression levels were calculated using EDGE-pro (Magoc et al., 2013) and Bowtie2 for mapping and alignment (Langmead and Salzberg, 2012). Normalized values are given in Fragments Per Kilobase-million (FPKM). Differential expression was calculated using log-ratios of FPKM values.

## Results and Discussion

### Progress in Analysis of the Operon Encoding the Dentilisin Protease Complex

Disruption of *Prt*P activity and/or its interaction(s) with *Prc*B and *Prc*A may be important factors in decreasing the virulence of *T. denticola* in its host-pathogen relationships. Understanding the mechanism(s) of these interactions may lead to more effective treatments of periodontal diseases. Our approach to analysis of the dentilisin operon focuses on construction and analysis of defined mutations within one or more genes of the operon. We refer reviewers to our published work for detailed presentation and analysis (Fenno et al., 1998; Lee et al., 2002; Bian et al., 2005; Godovikova et al., 2010; Godovikova et al., 2011; Goetting-Minesky et al., 2012). Key points are summarized below.

One of the first reported *T. denticola* defined mutants was reported by Ishihara et al. In this mutant strain, *prtP* was disrupted by insertion of an antibiotic resistance cassette, resulting in loss of dentilisin protease activity and attenuated virulence in a mouse abscess model (Ishihara et al., 1998). An unexpected result was disrupted expression of Msp, a highly expressed trimeric outer membrane protein encoded distally on the *T. denticola* chromosome. Subsequent studies by our group have generated more than a dozen defined polar and nonpolar mutations in the dentilisin locus. Phenotypes of these strains are listed in **Table 1**. As expected, any mutation that results in loss of PrtP expression abrogates protease activity, as measured by cleavage of the chromogenic substrate succinyl-L-alanyl-L-alanyl-L-prolyl-L-phenylalanine-*p*-nitroanilide (SAAPFNA). These studies contributed to characterization of PrtP-dependent post-translational processing of PrcA into two polypeptides designated PrcA1 and PrcA2 (Lee et al., 2002), identification of PrcB (Bian et al., 2005) and initial characterization of the individual roles of each protein/polypeptide in expression of the active dentilisin complex (Godovikova et al., 2010; Godovikova et al., 2011).

**Figure 2:**
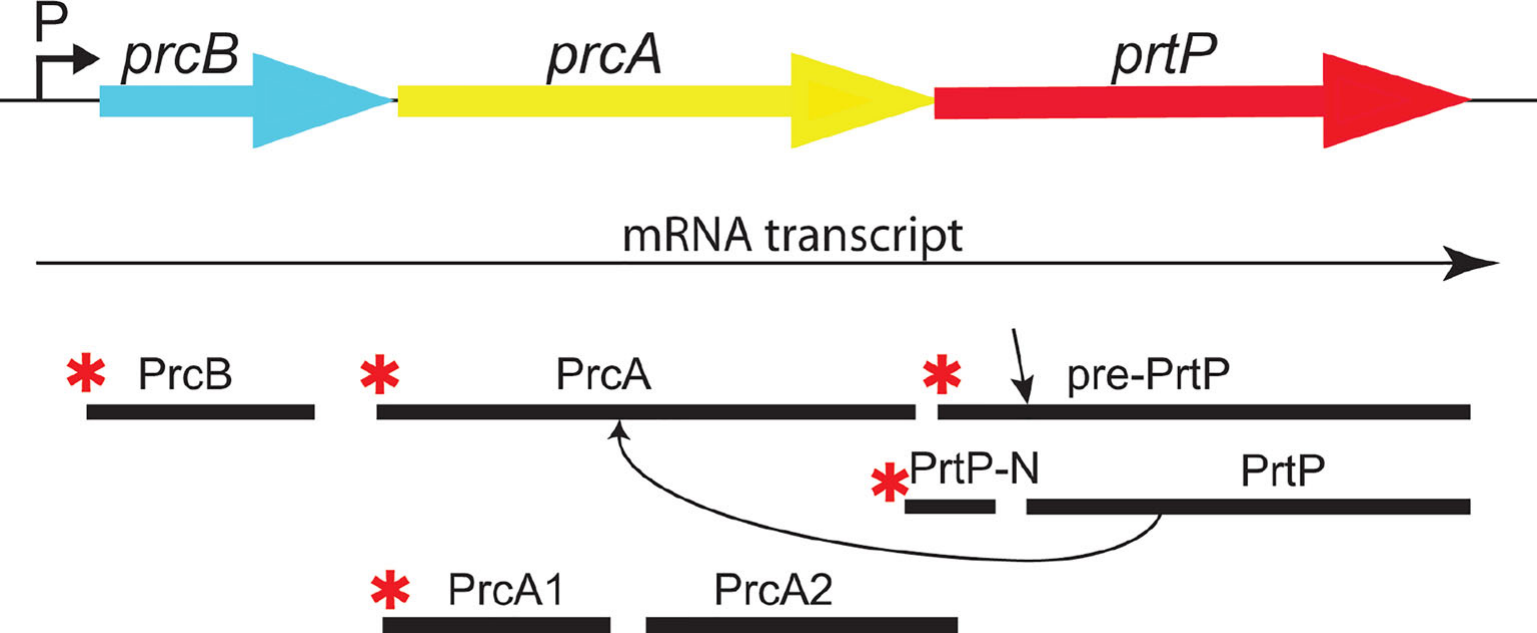
The dentilisin operon in *T. denticola* ATCC 35405. The three genes of the protease operon are expressed as a single mRNA transcript. PrtP is expressed as a preproprotein which is activated upon removal of the prepropeptide (N-PrtP). Asterisks (*) indicate predicted N-terminal acylation. PrtP is required for processing of PrcA to PrcA1 and PrcA2. [adapted and modified from (Godovikova, Goetting-Minesky et al. 2011)].

The first gene in the protease operon (**Figure 2**) encodes PrcB, a previously uncharacterized 23-kDa lipoprotein with no homologs in species other than oral *Treponema*. PrcB is required for expression of and interacts with PrtP, but is not required for PrcA expression (Bian et al., 2005). The second gene encodes PrcA, a 70-kDa lipoprotein that is cleaved to polypeptides (PrcA1, PrcA2) of approximately 40-kDa and 30-kDa representing the acylated N-terminal and non-acylated C-terminal PrcA domains, respectively (Lee et al., 2002). PrcA cleavage is dependent on PrtP proteolytic activity (Lee et al., 2002).

PrtP is a member of the widespread subtilisin (S8) family of proteases and peptidases (Ishihara et al., 1996; Donlon, 2007) that includes numerous prokaryotic enzymes (Siezen et al., 2007). Interestingly, the only other acylated subtilisin-like protease is the SphB1 autotransporter in *Bordatella* spp., which is involved in maturation and extracellular release of filamentous hemagglutinin (Coutte et al., 2003). PrtP is reported to be activated by cleavage after residue 158 (Tyr) by an as-yet unknown mechanism (Ishihara et al., 1996).

The dentilisin complex purified from *T. denticola* outer membrane extracts contains PrcB, PrcA1, PrcA2 and PrtP as a detergent-stable complex of approximately 100-kDa (Uitto et al., 1988; Godovikova et al., 2011). All four polypeptides are present in equimolar amounts in native dentilisin. Interestingly, the acylated 15-kDa PrtP N-terminal polypeptide resulting from PrtP activation is not found in the purified dentilisin complex but is present in membrane extracts of whole cells. It should be noted that, of the four polypeptides in the mature protease complex, only PrcA1 and PrcB retain their N-terminal lipid moieties, which presumably anchor the complex in the *T. denticola* outer membrane. We propose that PrcB and PrcA1 acyl moieties anchor the complex in the outer membrane, and PrcB-PrtP and PrcA-PrtP interactions stabilize the complex at the cell surface. While the specific composition of the acyl modifications on the dentilisin components has not been determined, they most likely consist of the typical tripalmitoyl-*S*-glyceryl-cysteine (Pam3Cys) lipid moiety found on other spirochetal lipoproteins (Kelesidis, 2014; Haake and Zuckert, 2018).

Immunofluorescence microscopy with rabbit IgG antibodies raised against recombinant versions of each of the 5 polypeptide products of the protease operon showed that PrcB, PrcA1 and mature PrtP are surface-localized on intact *T. denticola* 35405 (Godovikova et al., 2011). Both PrcA2 and the acylated PrtP-N polypeptide released by PrtP activation were detected only in permeabilized *T. denticola* 35405 cells. Interestingly, PrcA2 was readily detected on a defined Msp-deficient mutant strain. Furthermore, demonstration of co-immunoprecipitation of Msp and PrcA2 using either anti-Msp or anti-PrcA2 antibodies provide strong evidence of direct interaction between these proteins. Msp expression is greatly reduced and Msp oligomers are absent in all but one dentilisin mutant, suggesting that either protease activity or Msp interaction with PrtP, PrcA or PrcB is required for Msp oligomerization and stability. Only one dentilisin locus mutant (CF522; **Table 1**) expresses native levels of oligomeric Msp. In this strain carrying a non-polar deletion of *prcB*, PrcA is expressed as an intact 70-kDa protein and *prtP* is transcribed, but PrtP protein is not detected (Godovikova et al., 2010). These results provide important information on protease complex structure and the protein interactions required for its presentation and stability on the cell surface. Studies are in progress to further characterize the connections between expression of Msp and dentilisin.

### Conservation of PrtP

**Figure 3:**
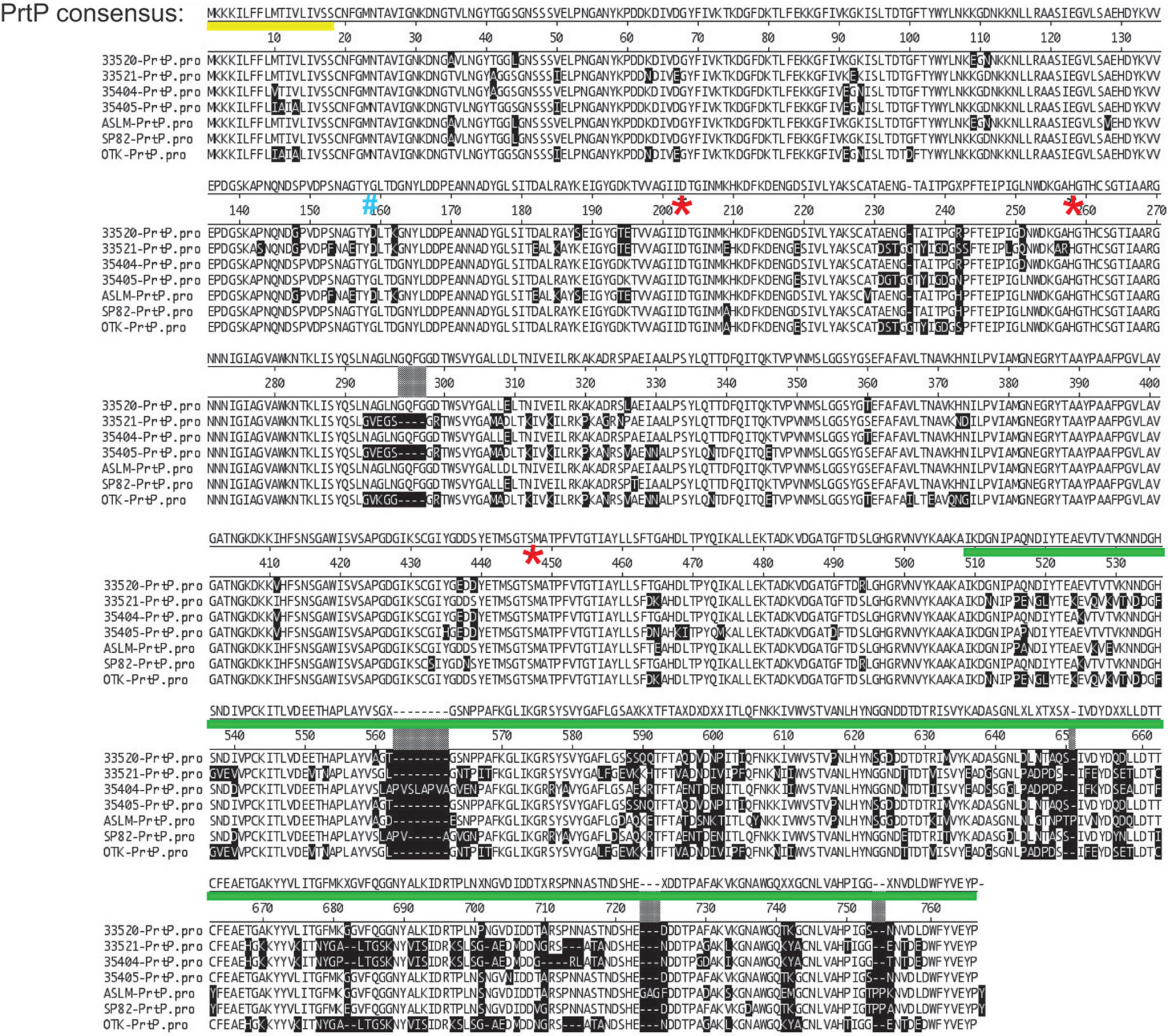
Alignment of PrtP’s of 7 *T. denticola* strains. Note that much of the strain variability is in the C-terminal region of PrtP. These differences may confer immunogenic variation between strains or may be associated with substrate specificity according to strain. The PrtP Type II signal peptide is indicated by a yellow line under the consensus sequence. The first residue of the mature protein (G^159^) is indicated by a blue “#.” Residues of the catalytic triad (Asp^203^, His^258^, and Ser^447^) are indicated by red asterisks. The PrtP C-terminal domain is indicated by a green line (adapted and modified from (Goetting-Minesky et al., 2012).

Initial annotation of the protease operon (especially *prcB* and *prtP*) in the *T. denticola* genome sequences in NCBI databases was inconsistent with our functional analysis. To this day, most databases contain errors in both PrcB and PrtP. The acylated N-terminal region of PrcB is missing and the C-terminus of PrtP contains a sequencing error that introduces a frameshift. Problematically, TDE0762 encoding PrtP is annotated as encoding a pseudogene. One serious consequence of this is that PrtP is not retrieved in searches based on protein homology or amino acid sequence. We analyzed the protease locus in seven diverse *Td* strains to [1] resolve anomalies in PrcB and PrtP sequence annotation and [2] determine the relationship between interstrain PrtP sequence heterogeneity and proteolytic activity (Goetting-Minesky et al., 2012). We resolved the *prcB* and *prtP* DNA sequences and identified prior sequencing and annotation errors (**Figure 3**). Subsequent to our publication, the pseudogene label was removed from the Human Oral Microbiome website (www.homd.org), but as on other genome websites, DNA sequences of *prcB* and *prtP* remain uncorrected. The corrected C-terminus of the PrtP protein was confirmed by construction of a *T. denticola* 35405 mutant strain carrying a C-terminal 6xHis tag on PrtP [(Goetting-Minesky et al., 2012) and **Table 1**]. Native PrtP-6xHis had wildtype dentilisin activity. Interstrain variability was highest in the C-terminal region of PrtP, while PrcB and PrcA were each highly conserved throughout their entire sequence (89% and 77% identity, respectively). The 508-residue PrtP N-terminal region that includes the catalytic domain were highly conserved in common laboratory strains and clinical isolates. While PrtP varied up to 20% between strains over the C-terminal 270 residues, the eight C-terminal -residues (DWFYVEYP) were present in all strains examined. Proteolytic activity varied considerably between strains (Goetting-Minesky et al., 2012). This may be due to interstrain differences in expression levels of the protease operon, which remain to be characterized in detail. The notable interstrain differences in the PrtP C-terminal domain suggest that it may be surface exposed and subject to antigenic pressure. This is supported by the observation that cell-surface immunoreactivity of antibodies raised against a PrtP polypeptide lacking the C-terminal domain is relatively weak compared with that of antibodies raised against the native dentilisin complex or other recombinant dentilisin components or the native dentilisin complex (Godovikova et al., 2011).

### Predicted Structure of PrtP

**Figure 4:**
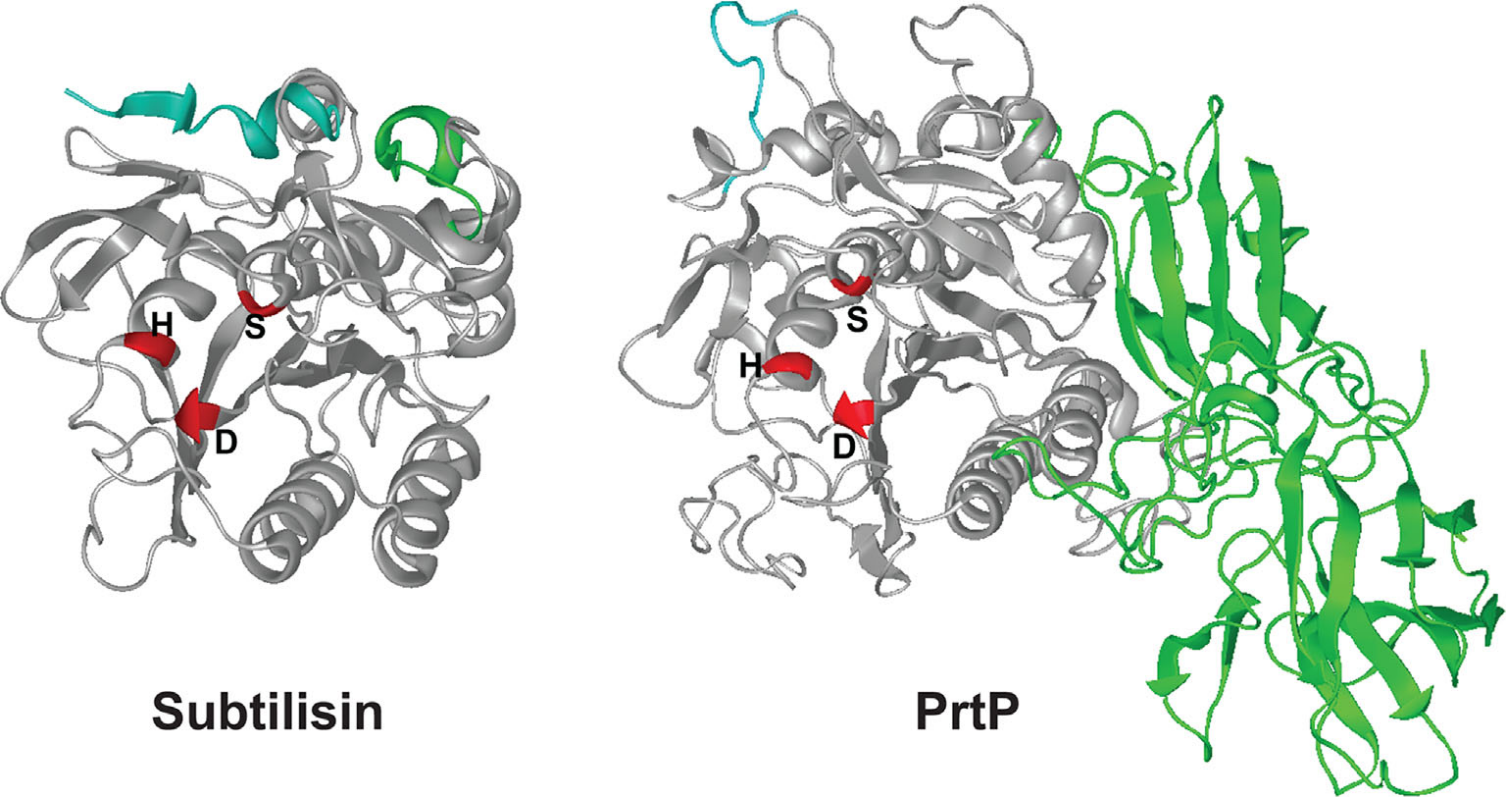
3-D structures for subtilisin and PrtP predicted by I-TASSER. Structures shown are the mature proteins (subtilisin, beginning at residue 207; PrtP, beginning at residue 159). The first ten residues of each protein are shaded blue for reference purposes only. Catalytic triad residues of each protein are shaded red. The C-terminal PrtP domain is shaded green. Note the strikingly similar configurations of the enzymes except for the C-terminal extension of PrtP. The function of this region is under investigation (https://zhanglab.ccmb.med.umich.edu/I-TASSER/).

PrtP has considerable homology with the *B. subtilis* serine protease subtilisin, and the catalytic triad of active-site residues (Asp^203^, His^258^, and Ser^447^) are conserved in the corresponding region of the PrtP protein ( (Ishihara et al., 1996) and **Figure 4**). We used the I-TASSER algorithm (Yang et al., 2015) to generate a predicted structure for PrtP and compared it to the known structure of subtilisin. The amino acid sequence of the mature, active PrtP protein (residues 159-766) was compared with that of mature subtilisin from *Bacillus subtilis* subsp. *subtilis* (GenBank: AEP90133.1; (Earl et al., 2012)). As shown in **Figure 4**, the predicted PrtP protein structure is very similar to that of subtilisin except for the N-terminus and residues 508-766. The PrtP N-terminus encodes a Type II signal peptide, which is not present in subtilisin. The entire PrtP C-terminal domain has no relationship with canonical subtilisins, which have molecular masses in the range of 28-kDa. The PrtP C-terminal domain shows considerable diversity among *T. denticola* strains, while the rest of the PrtP protein shows high interstrain homology [**Figure 3** and (Goetting-Minesky et al., 2012)]. The high level of interstrain variability suggests that the ~250-residue PrtP C-terminal domain is exposed to the extracellular environment, which could drive antigenic diversity between *T. denticola* strains, similar to what is seen in the Msp proteins of diverse *T. denticola* strains (Fenno et al., 1997; Godovikova et al., 2019).

### PrtP C-Terminal Domain Is Required for PrtP Expression

**Figure 5:**
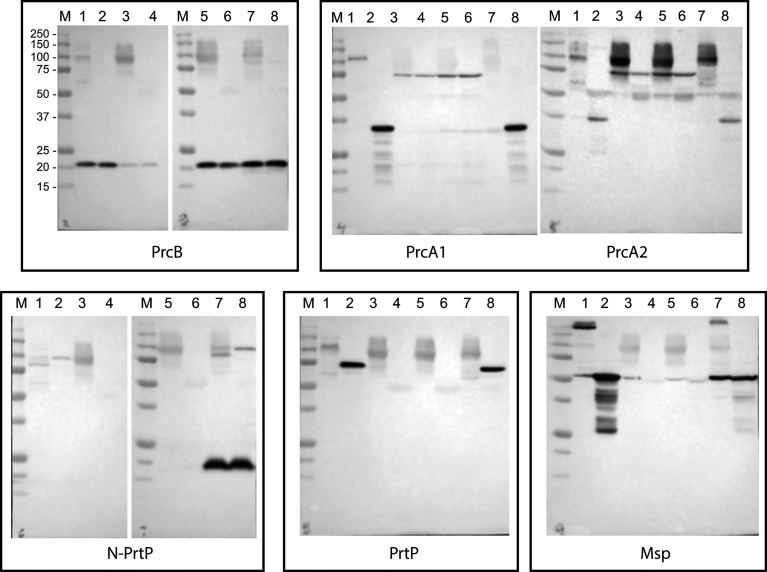
Western blot analysis of PrtP mutant strains. *T. denticola* lysates, either unheated (odd lanes) or boiled (even lanes), were probed with the indicated antibodies to components of the dentilisin complex and Msp. Lanes: 1 and 2: 35405; 3 and 4: CCE; 5 and 6: CF1010; 7 and 8: CF1031. Molecular weight standards for all blots are as indicated for the PrcB blot.

**Figure 6:**
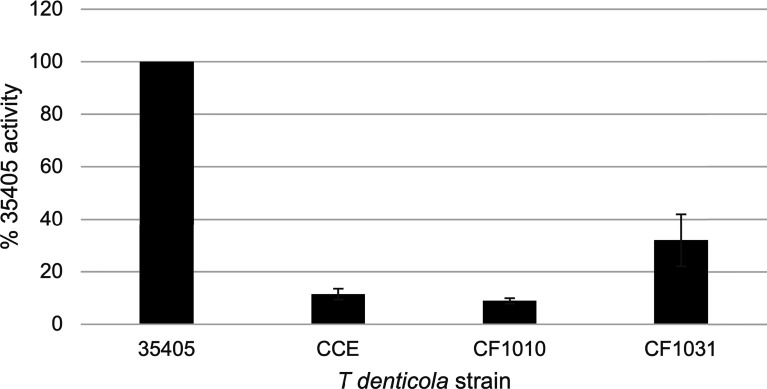
Proteolytic activity of *T. denticola* strains. *T. denticola* strains were assayed for ability to cleave the chromogenic substrate SAAPFNA. Protease activity of individual strains, assayed by change in absorbance at 405 nm, is expressed as a percentage relative to the SAAPFNA activity of strain 35405. Strains are as listed in **Table 1**. Error bars represent the standard error of three independent experiments with triplicate samples.

As noted above, the PrtP C-terminal domain is unique among subtilisin-like proteases and is unlikely to contribute directly to its proteolytic activity. To characterize the contribution of the PrtP C-terminal domain to expression of the dentilisin complex, we constructed an isogenic mutant in which DNA encoding the C-terminal 259 residues was replaced with gene cassette encoding erythromycin resistance (Goetting-Minesky and Fenno, 2010), yielding *T. denticola* strain CF1010 (**Table 1**). As shown in **Figure 5**, the PrtP protein was not detected in the mutant strain, suggesting that the C-terminal domain is required for proper translocation or folding of mature PrtP. DNA sequence analysis of the mutant strain showed no sequence anomalies in the remaining PrtP sequence (data not shown). Both PrcB and PrcA were expressed in CF1010. As observed in other mutants lacking in PrtP, PrcA was expressed as a 70-kDa protein and was not cleaved to polypeptides PrcA1 and PrcA2. This is consistent with previous studies (Lee et al., 2002) and supports the hypothesis that cleavage of PrcA is due to PrtP proteolytic activity. Consistent with the western blot analysis, *T. denticola* CF1010 had very little proteolytic activity, similar to that of *prtP* knockout strain CCE (**Table 1** and **Figure 6**). The very low level of SAAPFNA-degrading activity in these strains may be due to chymotrypsin-like activity of an unrelated minor *T. denticola* protease (Arakawa and Kuramitsu, 1994).

### Site-Directed Mutagenesis of PrtP Active Site

To further examine the role of PrtP proteolytic activity in expression and assembly of the dentilisin complex we constructed an isogenic *T. denticola* mutant carrying a Ser➔Ala mutation at residue 447, one of the three residues of the “catalytic triad” of subtilisin family proteases. To increase likelihood of recovering the desired mutation, we introduced the DNA fragment carrying the mutation and a Km^R^ cassette into the Em^R^ PrtP C-terminal deletion strain *T. denticola* CF1010. Following recovery and validation of the Km^R^/Em^S^ transformant CF1031, we analyzed dentilisin protease activity by SAAPFNA cleavage and protein expression by western blotting. While *T. denticola* CF1031 had greatly reduced SAAPFNA activity compared with strain 35405, its activity was higher than that of CF1010 or *prtP* knockout strain CCE (**Table 1** and **Figure 6**).

As shown in **Figure 5**, all three proteins of the dentilisin complex were expressed in CF1031, but with some unanticipated results. PrtP was expressed and migrated similarly in both 35405 and CF1031. As expected, PrcB was expressed as in the wild type strain. However, PrcA was cleaved to PrcA1 and PrcA2 as in 35405. Taken together, this suggests that mutagenesis of a single catalytic residue, while sufficient to abrogate most PrtP proteolytic activity, was insufficient to prevent cleavage of PrcA to PrcA1 and PrcA2, which we previously reported to be PrtP-dependent (Lee et al., 2002). It is possible that the Ser^447^➔Ala^447^ mutation was sufficient to block cleavage of SAAPFNA (**Figure 6**) but did not completely block PrtP activity. We plan further studies in which the Asp^203^ and His^258^ residues will be mutated.

Interestingly, the 15-kDa N-terminal polypeptide fragment of PrtP was detected at much higher levels in CF1031 than in 35405, indicating that activation of the zymogen form of PrtP is likely independent of its proteolytic activity. This is consistent with the activation pathway of other subtilisin-family proteins, in which activation and cleavage of the propeptide is driven by the propeptide itself (Shinde and Inouye, 2000; Subbian et al., 2005). Further studies characterizing activity of the PrtP propeptide (residues 19-158) are required to resolve this issue.

To our knowledge, this is the first report of site-directed single-base mutagenesis in *T. denticola.* In addition to its contribution to understanding the role of PrtP protease activity in post-translational processing and assembly of the components of the dentilisin complex, this mutant will be of particular utility in characterizing the mechanisms of dentilisin interactions with host cells and tissue components, as any effects would most likely be due to acylation. While most studies of dentilisin interaction with host cells and proteins focus on its protease activity (McDowell et al., 2011), there are ample reasons to hypothesize that dentilisin lipid moieties are also important in these processes. Spirochetes contain relatively high numbers of lipoproteins, and many have been shown to have pro-inflammatory or other immunomodulatory effects (reviewed in (Christodoulides et al., 2017; Haake and Zuckert, 2018)). *T. denticola* is reported to activate production of several inflammatory cytokines by a TLR2-dependent manner (Nussbaum et al., 2009; Ruby et al., 2018), but these studies implicated other *T. denticola* components. Most studies of dentilisin focus on its proteolytic activity. We are currently conducting studies on the interaction of dentilisin with TLR2 and its role in downstream intracellular response pathways (Ganther et al., doi.org/10.1101/2021.01.18.427101). Availability of a non-proteolytic, dentilisin-positive strain will permit analysis of the contribution of its considerable acyl components to *T. denticola-*host interactions.

### Transcriptomic Changes Resulting From Dentilisin Mutagenesis

To determine effects of global *T. denticola* gene transcription, we performed RNAseq analysis on *T. denticola* ATCC 35405 parent and P0760 strains (**Table 1**) after two days growth in broth culture, which represents early- to mid-logarithmic growth phase. We focused our attention on genes whose expression was most highly increased in the dentilisin mutant. As shown in **Table 2**, six of the twenty genes whose expression was most increased are annotated as being involved in iron metabolism, either through iron uptake (TDE1178-TDE1180, encoding an iron compound ABC transporter), iron sensing (TDE0163 and TDE1177, encoding flavodoxins) or as part of an iron-sulfur cluster (TDE1176). TDE1177-TDE1180, upregulated between 5.6- and 8.9-fold are predicted to comprise an operon. These results suggest that dentilisin protease activity may contribute to acquisition of iron-containing host proteins and that disruption of the protease operon may result in iron depletion or unavailability. One of the numerous host proteins that dentilisin is reported to degrade is transferrin (Mäkinen et al., 1995), which is an important mediator of host iron sequestration (Parrow et al., 2013). We are planning further exploration of the connection between dentilisin and *T. denticola* iron uptake and metabolism.

**Table 2:** RNAseq analysis of *T. denticola* dentilisin mutant P0760.

Gene ID	Product	Change (base 10)	Iron related
TDE0163	flavodoxin	8.9446	indicates iron limitation
TDE1180	iron compound ABC transporter periplasmic iron compound-binding protein	8.3191	iron transport
TDE1177	flavodoxin	8.0000	indicates iron limitation
TDE2524	ABC transporter ATP-binding protein	6.3125	
TDE0180	hypothetical protein	5.8384	
TDE1179	iron compound ABC transporter permease	5.7500	iron transport
TDE1176	oxygen-independent coproporphyrinogen III oxidase	5.6000	iron-sulfur cluster
TDE1511	pathogen-specific surface antigen	5.5314	
TDE2523	hypothetical protein	5.5000	
TDE1178	iron compound ABC transporter ATP-binding protein	4.9091	iron transport
TDE1517	hypothetical protein	4.3295	
TDE1524	hypothetical protein	4.3125	
TDE0181	methyl-accepting chemotaxis protein	4.2152	
TDE0942	long-chain-fatty-acid–CoA ligase	4.1742	
TDE1518	permease	4.1600	
TDE0040	AMP-binding protein	4.1579	
TDE1516	ABC transporter ATP-binding protein	3.8777	
TDE1515	permease domain protein	3.7708	
TDE1513	hypothetical protein	3.7523	
TDE1514	permease domain protein	3.7248	

The twenty most highly upregulated genes in T. denticola protease mutant P0760 (**Table 1**) compared with the parent strain, as detected by RNAseq analysis. Six of the top twenty genes in this group appear to be related to iron regulation. Fold change is based on comparison with expression of same genes in T. denticola ATCC 35405. As expected, expression of members of the protease operon was not detected in P0760.

Another predicted iron compound transporter, encoded by TDE0756-TDE0758 was expressed at approximately five-fold lower levels in the dentilisin mutant (data not shown). We hypothesize that this may be a result of the mutagenesis strategy used to construct strain P0760, in which the 5’ region of *prcB* was disrupted by insertional mutagenesis (Bian et al., 2005). The TDE0756-TDE0758 operon and the dentilisin operon (TDE0760-TDE0762) are encoded on opposite DNA strands, and their promoters overlap (data not shown). Further transcription studies in one of the dentilisin mutants in which the 5’end of the operon is intact will be required to resolve this issue.

### Summary

*T. denticola* dentilisin is a unique lipoprotein complex implicated in a wide range of interactions with host tissue, cells and circulating immunomodulatory components. By focusing on the molecular mechanisms of expression and assembly of dentilisin, we hope to contribute to understanding of its role in periodontal pathogenesis at a mechanistic level. In other ongoing and planned studies, we are utilizing the mutant strains described herein to characterize the contributions of dentilisin and its unique individual features, specifically its PrtP protease activity and the lipid components of all three proteins, to the observed effects of *T. denticola* in the subgingival oral environment.

## Author Contributions 

All authors contributed to data generation and analysis. JCF and MPG-M conceptualized and wrote the paper. All authors reviewed and edited the paper.

## Funding

This study was supported by Public Health Service grants DE025225 and DE018221 (to JCF) from the National Institute of Dental and Craniofacial Research.

## Conflict of Interest

The authors declare that the research was conducted in the absence of any commercial or financial relationships that could be construed as a potential conflict of interest.
